# Ytterbium-doped fiber laser passively mode locked by few-layer Molybdenum Disulfide (MoS_2_) saturable absorber functioned with evanescent field interaction

**DOI:** 10.1038/srep06346

**Published:** 2014-09-12

**Authors:** Juan Du, Qingkai Wang, Guobao Jiang, Changwen Xu, Chujun Zhao, Yuanjiang Xiang, Yu Chen, Shuangchun Wen, Han Zhang

**Affiliations:** 1 Key Laboratory for Micro-Nano Optoelectronic Devices of Ministry of Education, College of Physics and Microelectronic Science, Hunan University, Changsha 410082, China; 2 Key Laboratory of Optoelectronic Devices and Systems of Ministry of Education and Guangdong Province, College of Optoelectronic Engineering, Shenzhen University, Shenzhen 518060, China

## Abstract

By coupling few-layer Molybdenum Disulfide (MoS_2_) with fiber-taper evanescent light field, a new type of MoS_2_ based nonlinear optical modulating element had been successfully fabricated as a two-dimensional layered saturable absorber with strong light-matter interaction. This MoS_2_-taper-fiber device is not only capable of passively mode-locking an all-normal-dispersion ytterbium-doped fiber laser and enduring high power laser excitation (up to 1 W), but also functions as a polarization sensitive optical modulating component (that is, different polarized light can induce different nonlinear optical response). Thanks to the combined advantages from the strong nonlinear optical response in MoS_2_ together with the sufficiently-long-range interaction between light and MoS_2_, this device allows for the generation of high power stable dissipative solitons at 1042.6 nm with pulse duration of 656 ps and a repetition rate of 6.74 MHz at a pump power of 210 mW. Our work may also constitute the first example of MoS_2_-enabled wave-guiding photonic device, and potentially give some new insights into two-dimensional layered materials related photonics.

Two-dimensional layered materials are considered as promising building blocks for the next-generation photonics technology, owing to its unique planar advantages^1^,^2^,^3^. Particularly, the existence of quantum confinements, the absence of inter-layer interactions and the strong intra-layer covalent or ionic bonding, allow researchers to fabricate compact, functional, flexible and efficient photonic devices. Graphene, the most representative two-dimensional nano-materials with Dirac-like electronic band structure, has become one of the most heavily studied targets among optical researchers in the past few years^4^,^5^,^6^,^7^, which are encouraged by some unique optical advantages in graphene, such as ultra-fast photo-response and ultra-wideband response ranging from ultraviolet (UV) to terahertz^8^. In spite of those merits, unfortunately, graphene holds two intrinsic disadvantages, the zero band gap and the weak absorption co-efficiency (~2% of incident light per layer) that significantly delimit its light modulation ability and potential applications in optics related fields that may require strong light-matter interaction.

In addition to carbon-based two-dimensional materials, atomic layered transition-metal dichalcogenides (TMDs) are now under continuously rising attentions due to their exceptional optical properties that may complement with graphene and other two-dimensional crystals (such as insulating hexagonal boron nitride), and even over-come the above-mentioned disadvantages of graphene. Molybdenum disulfide (MoS_2_), one typically shining material of TMDs, possesses a thickness dependent electronic and optical property. That is, bulk MoS_2_ has an indirect band-gap with weak light-matter interaction while mono-layer or few-layer MoS_2_ turn out to be a direct band-gap semiconductor with enhanced light activity^9^. Its thickness dependent band-gap and electronic band structure endow it with many new optical properties that are distinct from graphene^10^,^11^,^12^,^13^. Determined by the unique symmetry of its lattice structure, few-layer MoS_2_ shows an interesting layer dependent^14^,^15^ or orientation dependent second order optical nonlinearity^16^, which is fundamentally different from graphene which possesses very weak second order nonlinearity. Concerning its third order nonlinear optical response, worthy of mentioning is that according to recent findings by various research groups, few-layer MoS_2_ had been ambiguously verified to exhibit enhanced optical saturable absorption (that is the imaginary part of the third order nonlinear susceptibility, corresponding to the optical absorption that decreases with the increase of the incident optical power and becomes saturated once the optical power reaches the threshold), due to its semi-conducting property^17^,^18^.

The transient absorption study by Cui *et al*. indicates that monolayer TMDs owns two types of excitation lifetimes (18 ± 1 ps and 160 ± 10 ps, respectively)^19^, indicating that monolayer TMDs can be developed as a new saturable absorber with two different relaxation time that can fit for specific applications. The enhanced, broadband and ultra-fast nonlinear optical property in few-layer TMDs suggests some unique potential for ultra-fast photonics, ranging from high-speed light modulation, ultra-short pulse generation to ultra-fast optical switching^17^,^18^. However, according to an experimental investigation on laser thinning of few-layer MoS_2_ (even under a power density of mW/μm^2^) by Castellanos-Gomez *et al.*^20^, the stability and robustness issues of TMDs becomes a significant problem if exposed to high power laser illumination. Unlike graphene that has extremely high thermal conductivity, flexibility and mechanical stability, TMDs may show much lower optical damage threshold than graphene because of their poorer thermal and mechanical property, although explorations on the photonic applications are being fueled by their advantages.

Naturally, optical damage gradually turns out to be a bottleneck in few-layer TMDs related optical applications, particularly towards high power regime. Consequently, it becomes very urgent to develop a scheme circumventing the optical damage of TMDs. Here, the “lateral interaction scheme” was employed to boost the optical damage threshold of few-layer MoS_2_, which was composed by thermally fragile van der Waals stacking of the covalently bonded S–M–S layers. Unlike perpendicular illumination upon the surface of few-layer MoS_2_^20^, where the laser beam is focused to a size of micro-meter and therefore extra heating could not be rapidly dissipated, lateral interaction between few-layer MoS_2_ and evanescent field (for example, the side-polished or tapered fiber) allows it to survive with an incident power higher than 1 W, which further guarantees the reliable nonlinear operation against thermal damage. Our nonlinear optical characterization measurement shows that this device exhibits polarization dependent saturable absorption response, which may be related with the polarization sensitive light-matter interaction between light and few-layer MoS_2_. The center wavelength, spectral width, repetition rate, and estimated pulse duration of the resultant MoS_2_-based passively mode-locked fiber laser are 1042.6 nm, 8.6 nm, 6.74 MHz, and 656 ps at a pump power of 210 mW, respectively. The intra-cavity pulse energy can reach up to 3.1 nJ.

## Results

### Characteristics of MoS_2_ samples

**Figure 1:**
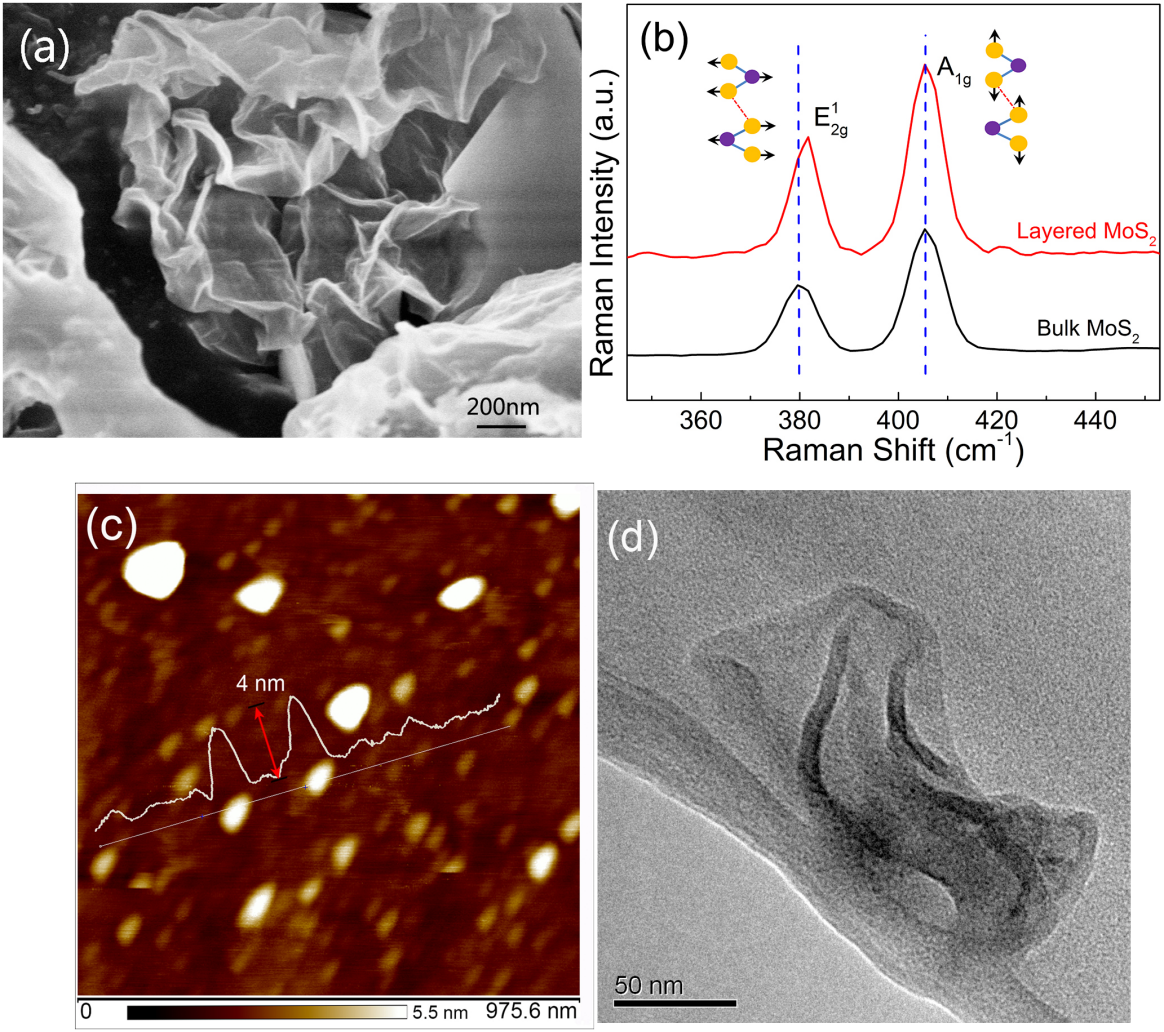
Characterization of the as-prepared few-layer MoS_2_. (a) Scanning electron microscopy image; (b) the Raman characterization; (c) the atomic force microscopy (AFM) characterization and (d) the transmission electron microscope (TEM) characterization.

The characterizations of the few-layer MoS_2_ samples, which are fabricated through the conventional Hydrothermal intercalation/exfoliation approach previously highlighted^21^,^22^, are summarized in Fig. 1. The scanning electron microscopy (SEM) image of the sample (Fig. 1a) shows the layered structure at the edge of the nano-plate. Further Raman Characterization shows that the *E*^1^_2g_ Raman peak (an in-plane motion of Molybdenum and Sulfide atoms) red shifts after complete exfoliation, indicating that the few-layer MoS_2_ with thicknesses in the range of 1–3 layers had been successfully fabricated^23^. Further AFM and TEM characterizations also confirmed its few-layer structure (Fig. 1c, d).

### Characteristics of MoS_2_ based saturable absorber

**Figure 2:**
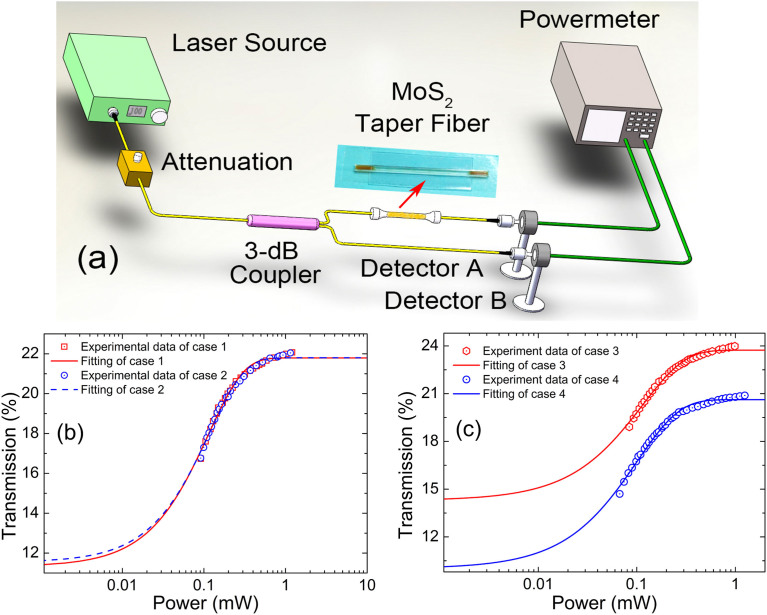
Characterization of the saturable absorption property of the MoS_2_-taper-fiber device. (a) Schematic diagram of the nonlinear optical characterization experiment setup. (b) The twin-detector measurement of the MoS_2_-taper-fiber device at 1041.3 nm. (c) Polarization dependent twin-detector measurements.

After the successful fabrication of few-layer MoS_2_, we then consider to fabricate few layer MoS_2_ based optical device. One of the most convenient ways is to deposit few-layer MoS_2_ onto the tapered fiber. The tapered section of an optical fiber is placed into a semicircular tube and fixed by adhesive dropped at the end, as shown in the photo of Fig. 2a. In the following, we designed a balanced twin-detector measurement system to investigate the nonlinear optical absorption characteristics of the as-fabricated saturable absorber. The laser source is a home-made pico-second Ytterbium doped fiber laser (repetition rate: 6.54 MHz and central wavelength: 1041.3 nm). By gradually changing the input power, a series of optical transmittance with respect to different input intensities had been recorded. Then, by fitting the relation between the optical transmission and the input laser power by using the following formula: *T*(I) = 1 − ΔT * exp(−I/I*_sat_*) − T*_ns_* where, *T*(I) is the transmission rate, ΔT is the modulation depth, I is the input intensity, I*_sat_* is the saturating intensity, and T*_ns_* is the non-saturable absorbance, the corresponding nonlinear optical parameters could be characterized. In order to further confirm that the nonlinear optical response is intrinsically caused by the sample itself other than some artifices, the optical transmission data had been measured by using two different cases, that is, the input power is increased from low to high power regime (case 1, the modulation depth: 10.47% and the saturable power: 0.88 mW) or vice versa (case 2, the modulation depth: 10.27% and the saturable power: 0.86 mW). Both curves overlap in a reasonable way, indicating that this saturable absorber component shows good stability with high optical damage threshold. This MoS_2_-taper-fiber device also shows polarizing effect, similar to the operation mechanism of graphene polarizer^33^. The mutual interaction of light propagating along the intra-layer of MoS_2_ allows for the emergence of polarization dependent nonlinear optical response. Experimentally, two orthogonally polarized laser beams were subsequently used to illuminate the MoS_2_-taper-fiber device, and therefore its polarization dependent saturable absorption properties can be identified. Interestingly, we noted that each polarization gives different saturable absorption parameters, that is, under horizontal polarization (case 3); the modulation depth and the saturable power are 9.4% and 0.87 mW, respectively. However, the modulation depth and the saturable power are found to be 10.61% and 0.99 mW, respectively, for the vertically polarized light (case 4). It suggests that our MoS_2_ based optical device not only functions as a saturable absorber, but also shows a weak polarizer, leading to some novel MoS_2_ enabled polarization devices, such as, 2-Dimensional layered polarizer, polarization controllers and so on.

### Mode locked fiber laser with MoS_2_ based saturable absorber

**Figure 3:**
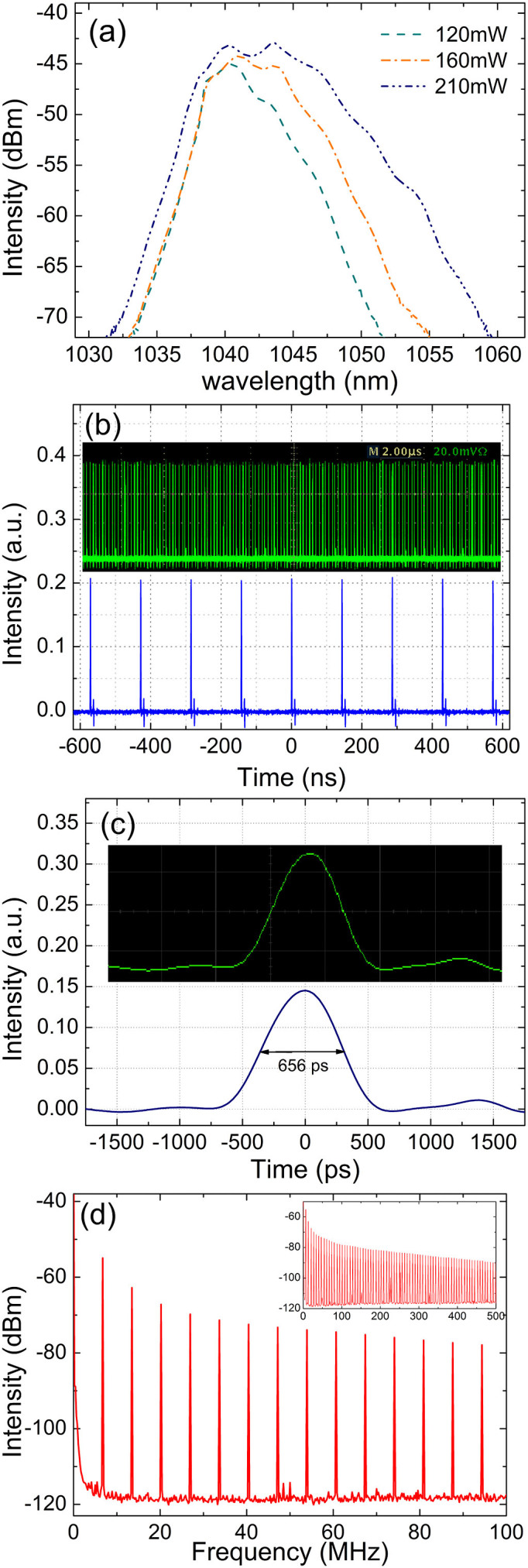
The laser mode-locking performance by the MoS_2_-taper-fiber saturable absorber. (a) Optical spectra of the generated dissipative solitons under different pump powers; (b) the wide-band oscilloscope tracings; (c) the individual pulse profile; (d) the radiofrequency spectral profile and insert: the wideband RF spectrum.

We designed a fiber laser cavity schematically at wavelength of 1 μm in order to evaluate its mode-locking ability. Under a pump power of 120 mW, which corresponds to the mode-locking threshold of the current laser cavity, the operation of stable mode-locked pulse can be readily obtained provided that the intra-cavity polarization controllers are suitably adjusted. The relatively high mode-locking threshold was caused by the high insertion loss of the saturable absorber. The optical spectra measured under a pump power of 120 mW, 160 mW and 210 mW, respectively were summarized in Fig. 3 (a). The optical spectra broaden with the increase of the pump power. At a pump power of 210 mW, its 3-dB spectral bandwidth is measured to be about 8.6 nm (with central wavelength located at 1042.6 nm). Fig. 3 (b) shows a stable mode-locked pulse train with a repetition rate of 6.74 MHz, which matches with the cavity length (~30.7 m). Its long range (up to 20 μs) stability had been characterized by the insert of Fig. 3 (b). The mode-locked pulse has pulse duration of 656 ps, indicating that the optical pulse is heavily chirped with a time-bandwidth product up to 1557. The large frequency chirp is also a characteristic of dissipative soliton, whose formation is a natural consequence of the mutual balance between the cavity gain, loss, dispersion and nonlinearity. Its formation mechanisms had also been widely investigated in graphene mode-locked dissipative soliton fiber lasers^34^,^35^,^36^,^37^,^38^,^39^. The corresponding narrow-band (0 ~ 100 MHz) and wideband (0 ~ 500 MHz) radio frequency spectrum shows that our laser cavity operates at the stable regime, given that the fundamental frequency (~6.74 MHz) has a high signal-to-noise ratio (up to 59 dB).

**Figure 4:**
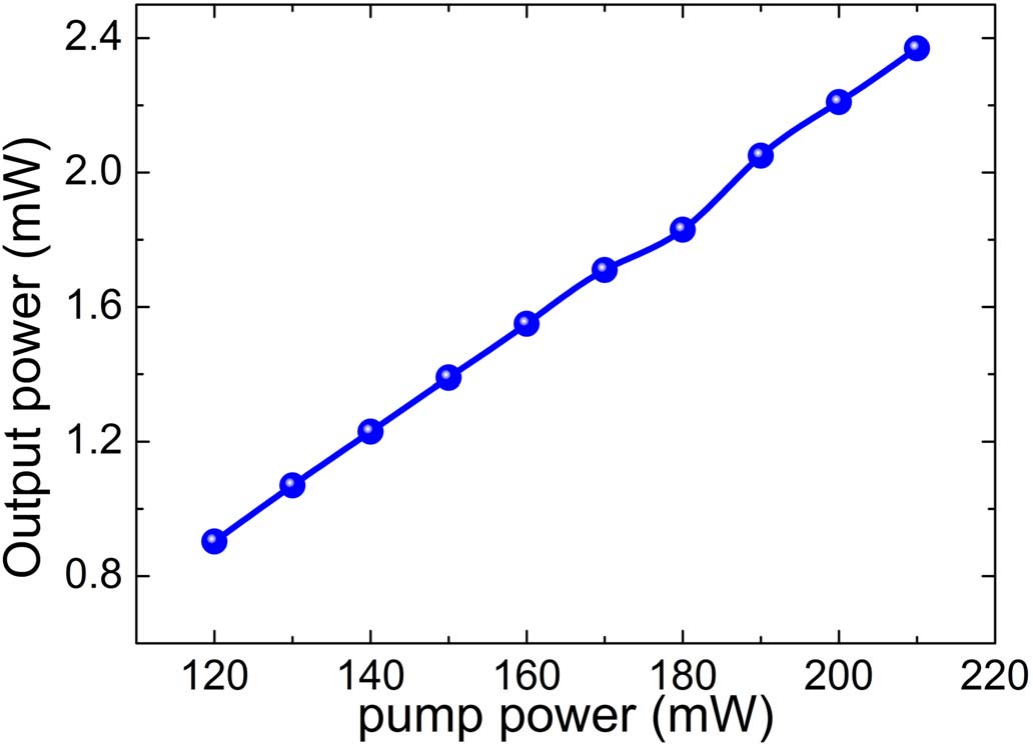
The relation between the input pump power and output power.

**Figure 5:**
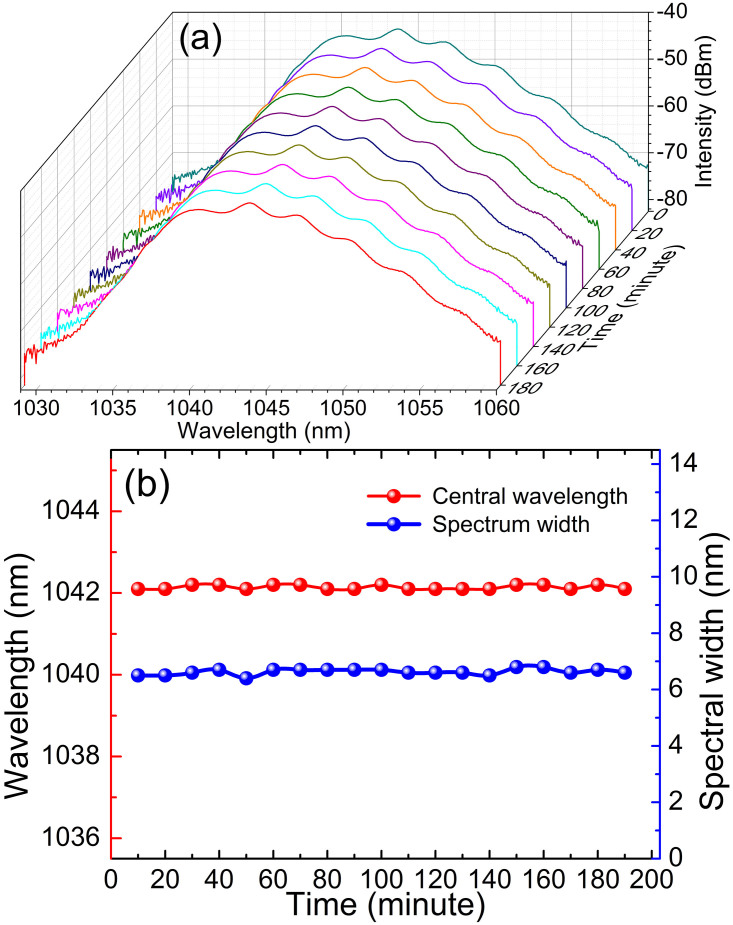
Long-term stability of the mode-locked dissipative soliton. (a) Repeated optical spectra measured every 20 minutes. (b) The drift of the central wavelength and the spectral bandwidth with respect to time.

The relation between the input pump power and output power is also characterized in Fig. 4, from which it is clear to note that the output power increases from 0.9 mW to 2.37 mW with the corresponding pump power increasing from 120 mW to 210 mW. The optical-to-optical efficiency is relatively low (only 1.1%) because of the high insertion loss from the tapered fiber. Further work by using tapered fiber with lower insertion loss can benefit for the high power operation. In order to examine the long term stability of the mode-locked lasers, we continuously monitor the output spectra of the mode-locked pulse for 3-hours. Fig. 5 shows the evolution of the central wavelength and the spectral bandwidth, where the central wavelength only changes from 1042.1 nm to 1042.2 nm while its 3-dB bandwidth fluctuates from 6.4 nm to 6.7 nm, further indicating the stability of the mode-locked fiber laser.

**Figure 6:**
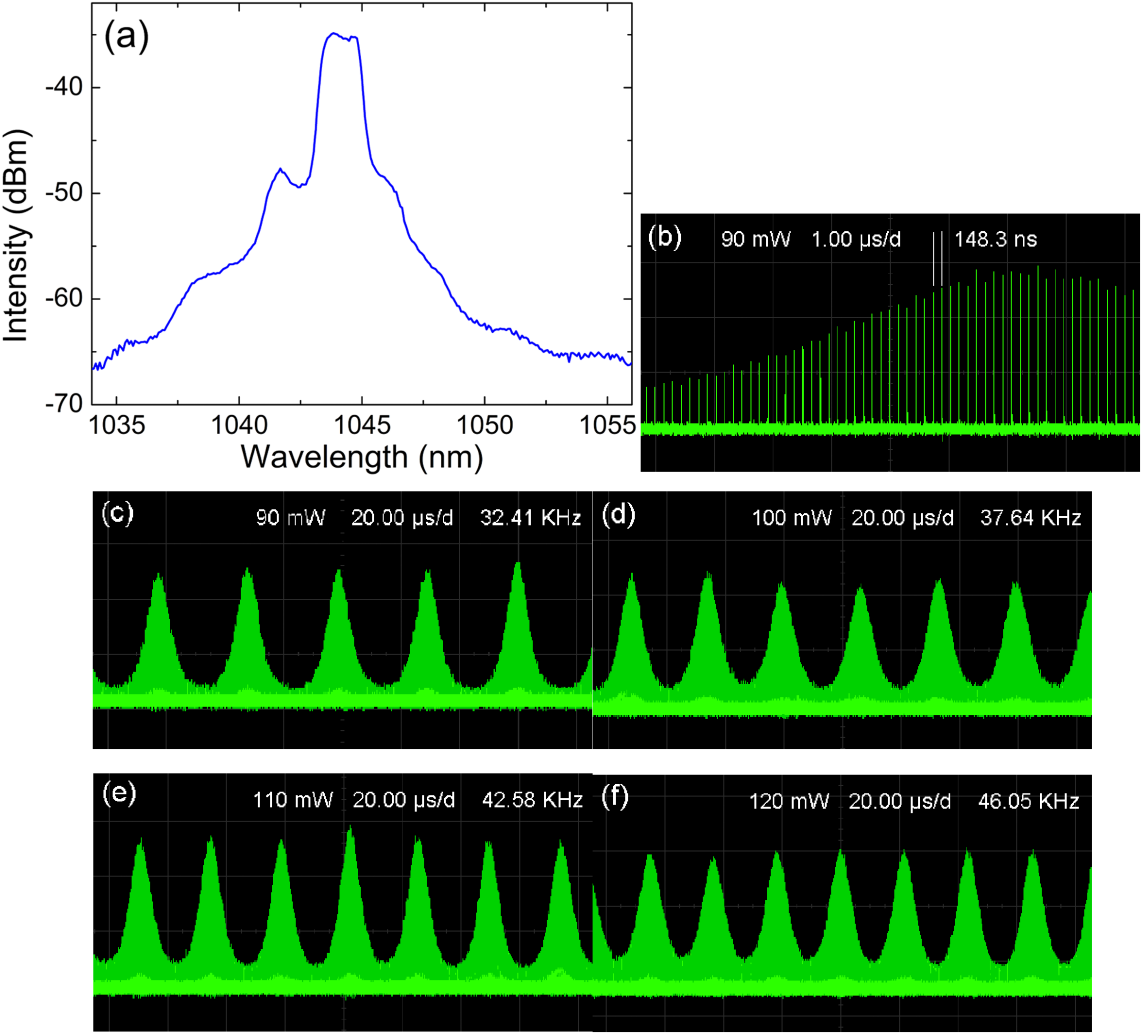
The Q-switched mode-locking operation. (a) The optical spectrum (b) the oscilloscope trace in microsecond scale and (c) in millisecond scale. (c)–(f) Different Q-switched mode-locking operation states under different pumping strength.

At a lower pump power, the operation of continuous-wave mode-locking may lose its stability while the Q-switched mode-locking operation occurs instead, as shown in Fig. 6. At a pump power of 90 mW, the optical spectra and oscilloscope traces are summarized in Fig. 6 (a) and Fig. 6 (b, c), respectively. The envelope of the Q-switched mode-locked pulse has a repetition rate of 32.41 kHz while the internal pulse-to-pulse separation remains the same as the cavity length, which is a typical characteristic of the Q-switched mode-locking operation. By controlling the cavity loss through over bending the intra-cavity fiber loop or rotating the polarization controllers, the repetition rate of the Q-switched mode-locking pulse train could be largely tuned, as shown in Fig. 6 (d–f), where the pulse train has a repetition rate of 37.64 kHz, 42.58 kHz, and 46.05 kHz, respectively.

**Figure 7:**
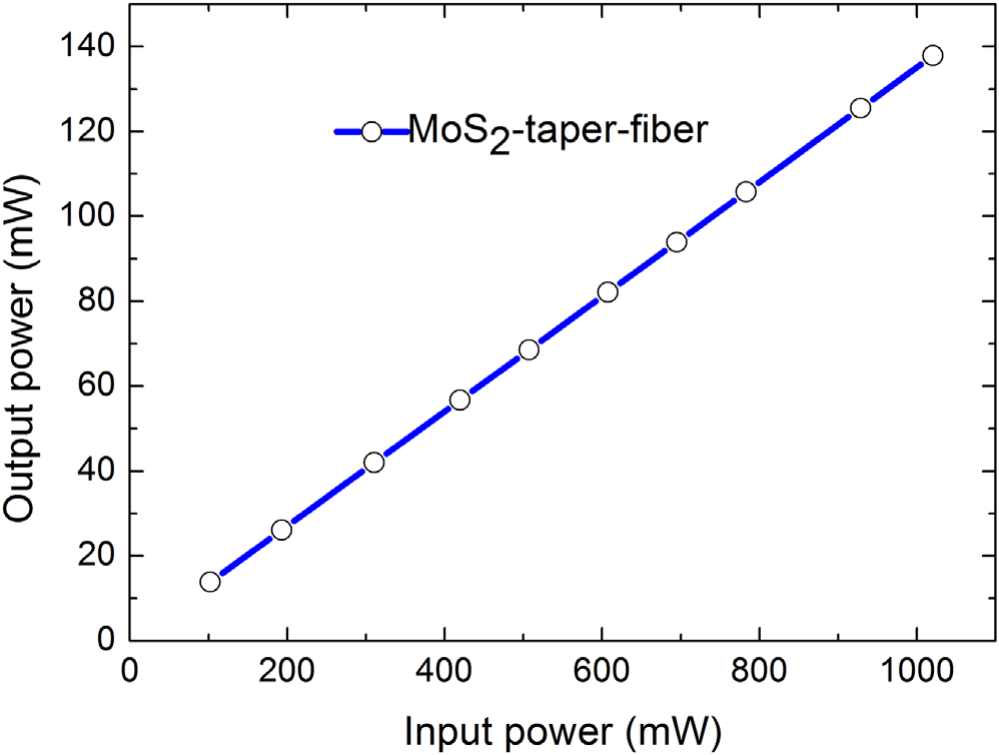
Laser-Induced Damage Threshold (LIDT) testing on this MoS_2_-taper-fiber device at 1064 nm.

Finally, in order to verify whether this device can endure high power, we also perform the laser-induced damage threshold (LIDT) experiment to check its damage threshold. In the experiment, a weak CW at 1064 nm was firstly amplified through a high power Ytterbium doped fiber amplifier (CYFA-PB-BW1-SM-42-NL0-OM1-B301-FA-FA) and then directly connected with this MoS_2_-taper-fiber device. Through monitoring the input and output power from this device, we note that its stability can be maintained under a laser power up to 1 W without optical damage, as shown in Fig. 7. By further introducing this device into the same laser cavity, the mode-locking performance could be still guaranteed without significant changes in terms of optical spectrum, pulse duration and optical loss, indicating that the as-fabricated device did not encounter degradation despite of high power illumination.

## Discussion

By depositing few-layer MoS_2_ upon the tapered fiber, we can employ a “lateral interaction scheme” of utilizing the strong optical response of few-layer MoS_2_, through which not only the light-matter interaction can be significantly enhanced owing to the long interaction distance, but also the drawback of optical damage of MoS_2_ can be mitigated. This MoS_2_-taper-fiber device can withstand strong laser illumination up to 1 W. Considering that layered TMDs hold similar problems as MoS_2_, our findings may provide an effective approach to solve the optical damage problem on those layered semiconductor materials. To take advantage of both saturable absorption and polarization sensitive absorption properties of this device, we have achieved the dissipative soliton mode locking operation with an optical pulse centered at 1042.6 nm. The passive Q-switched mode-locking operation further verifies the mode locking ability of MoS_2_. Beyond MoS_2_, we anticipated that a number of MoS_2_-like layered TMDs (such as, WSe_2_, MoSe_2_, TaS_2_ *etc*) can also be developed as promising optoelectronic devices with high power tolerance, offering inroads for more practical applications, such as large energy laser mode-locking, nonlinear optical modulation and signal processing *etc*.

## Methods

### MoS_2_-taper-fiber based saturable absorber fabrication

One of the most convenient ways of utilizing the light-matter interaction of MoS_2_ is to deposit few-layer MoS_2_ onto the tapered fiber, where evanescent light field propagates outside the fiber core. This method of taking advantage of both the saturable absorption of optical absorbing materials and the long interaction distance had been widely employed for fabricating different types of saturable absorber devices ranging from carbon nanotube^24^, graphene^25^,^26^,^27^ to topological insulator^28^,^29^,^30^,^31^,^32^. Through drop casting few-layer MoS_2_ solution onto the tapered fiber, MoS_2_-taper-fiber based saturable absorber device can be developed.

### Mode-locking experiment

**Figure 8:**
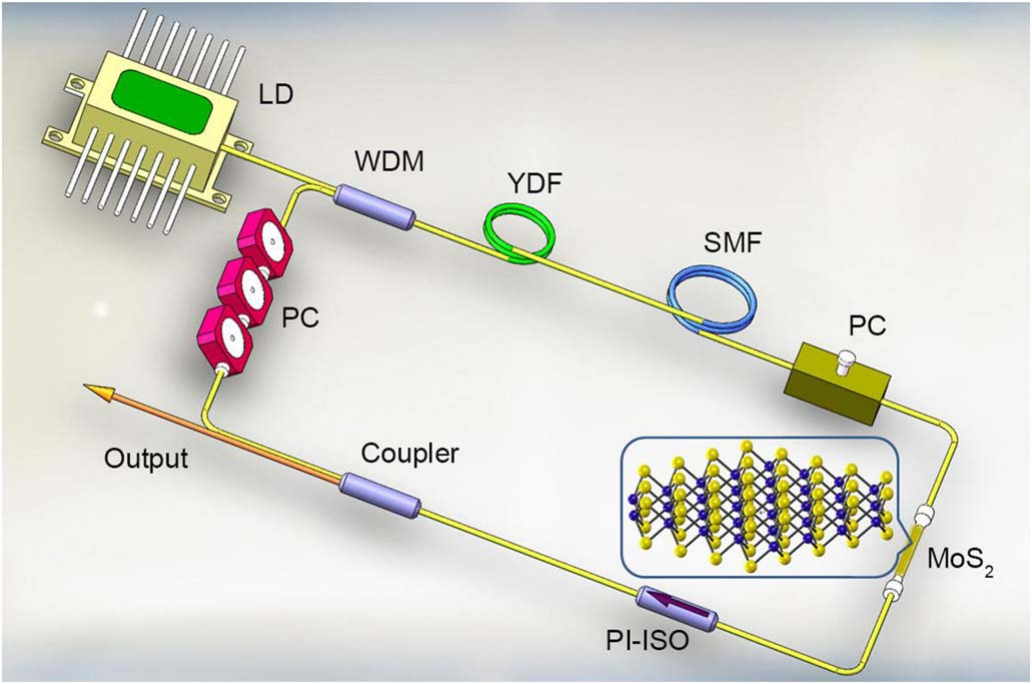
Schematic of the ytterbium-doped fiber laser passively mode locked by the MoS_2_-taper-fibersaturable absorber. WDM (wavelength division multiplexer), YDF (ytterbium-doped fiber), PC (polarization controller), PI-ISO (polarization-independent isolator), SMF (single-mode fiber) and MoS_2_-taper-fiber saturable absorber.

We designed a fiber laser cavity schematically shown in Fig. 8 in order to evaluate its mode-locking ability. Within the laser cavity, it consists of a piece of 0.65-m highly ytterbium-doped fiber (YDF, LIEKKI Yb1200-4/125) with a group velocity dispersion of 24.22 ps^2^/km as the gain medium, and a single-mode fiber (HI 1060) with a total length of 30.05-m fiber and group velocity dispersion of 21.91 ps^2^/km as the other fiber component. The net cavity dispersion was normal with a value estimated to be 0.68 ps^2^. A 975-nm laser diode was connected by a 975/1060 WDM and the pumping laser was therefore directed into the laser cavity. An in-cavity polarization-independent isolator (PI-ISO) was placed inside the laser cavity to ensure the unidirectional operation. The intra-cavity polarization controllers (PCs) were employed to adjust the cavity polarization and birefringence in order to optimize the laser mode-locking performance. The MoS_2_ saturable absorber device was spliced inside the laser cavity to start the mode-locking operation. The output laser pulse train, which could be transmitted outside the laser cavity through a 10/90 output coupler, was simultaneously monitored by an oscilloscope (Tektronix TDS3054B) and a 7 GHz radiofrequency analyzer (Agilent N9322C), while the optical spectra were measured by an optical spectrum analyzer (Ando AQ-6317B) with a spectral resolution of 0.015 nm. The center wavelength, average output power and estimated pulse duration of the resultant MoS_2_-based passively mode-locked fiber laser are 1042.6 nm, 2.37 mW and 656 ps at a pump power of 210 mW, respectively.

## Author Contributions

J.D., S.W. and H.Z. designed the experiment and wrote the paper. Q.W., G.J. and C.X. prepared the sample and performed the material characterization. C.Z., Y.X. and Y.C. performed the laser experiment. H.Z. supervised the project. All authors discussed the results and commented on the manuscript.
